# Role of valence changes and nanoscale atomic displacements in BiS_2_-based superconductors

**DOI:** 10.1038/srep37394

**Published:** 2016-11-22

**Authors:** Jie Cheng, Huifei Zhai, Yu Wang, Wei Xu, Shengli Liu, Guanghan Cao

**Affiliations:** 1 Center of Advanced Functional Ceramics, College of Science, Nanjing University of Posts and Telecommunications, Nanjing, Jiangsu 210023, China; 2 Department of Physics, Zhejiang University, Hangzhou 310027, China; 3 Shanghai Synchrotron Radiation Facility, Shanghai Institute of Applied Physics, Chinese Academy of Sciences, Shanghai 201204, China; 4 Beijing Synchrotron Radiation Facility, Institute of High Energy Physics, Chinese Academy of Sciences, Beijing 100049, China; 5 Rome International Center for Materials Science, Superstripes, RICMASS, via dei Sabelli 119A, I-00185 Roma, Italy; 6 Nanjing University (Suzhou) High-Tech Institute, Suzhou, 215123, China

## Abstract

Superconductivity within layered crystal structures has attracted sustained interest among condensed matter community, primarily due to their exotic superconducting properties. EuBiS_2_F is a newly discovered member in the BiS_2_-based superconducting family, which shows superconductivity at 0.3 K without extrinsic doping. With 50 at.% Ce substitution for Eu, superconductivity is enhanced with Tc increased up to 2.2 K. However, the mechanisms for the *T*_c_ enhancement have not yet been elucidated. In this study, the Ce-doping effect on the self-electron-doped superconductor EuBiS_2_F was investigated by X-ray absorption spectroscopy (XAS). We have established a relationship between Ce-doping and the *T*_c_ enhancement in terms of Eu valence changes and nanoscale atomic displacements. The new finding sheds light on the interplay among superconductivity, charge and local structure in BiS_2_-based superconductors.

Superconductivity in quasi-two-dimensional crystal structures has attracted sustained interest in the past decades. The most outstanding examples include high-*T*_c_ cuprates with CuO_2_ superconducting layers^1^ and Fe-based superconductors with a Fe-square lattice^2^. Very recently, superconductivity of BiS_2_-based compounds which have similar layered crystal structure as those of cuprates and Fe-based materials has been reported. The first member of the BiS_2_-based superconducting family is Bi_4_O_4_S_3_ with a *T*_c_ of 8.6 K^3^. It was found that the characteristic BiS_2_ layers are responsible for the superconductivity^3^. So far, several ReBiS_2_O_1-*x*_F_*x*_ (Re = La, Ce, Pr and Nd) and doped SrBiS_2_F superconductors have been discovered with the highest *T*_c_ of 10.6 K^4^,^5^,^6^,^7^,^8^,^9^,^10^. Band structure calculations indicate that the undoped parent compounds such as LaBiS_2_O and SrBiS_2_F are insulators with an energy gap of 0.82 and 0.80 eV, respectively^11^,^12^. Upon electron doping, both compounds exhibit metallic conducting behavior and a superconducting transition at low temperatures^4^,^10^. On the other hand, recent works demonstrate that the isostructural compounds EuBiS_2_F and Eu_3_Bi_2_S_4_F_4_ are metallic, and they even exhibit superconductivity without extrinsic doping, at temperatures below 0.3 K and 1.5 K respectively^13^,^14^, different from the other analogues. By various experimental approaches, it is pointed out that the self-doping nature of the observed superconductivity in both EuBiS_2_F and Eu_3_Bi_2_S_4_F_4_ is due to the mixed valence of Eu^13^,^14^. Currently, with 50 at.% Ce substitution for Eu in EuBiS_2_F, the *T*_c_ is enhanced up to 2.2 K^15^. It was suggested that the Eu valence is essentially divalent in Ce-doped system^15^. On the contrary, the average Eu valence with respect to the parent compound increases with the Se doping in Eu_3_Bi_2_S_4-*x*_Se_*x*_F_4_ which has the highest *T*_c_ of 3.35 K^16^. How the Eu valence changes and its consequence on superconductivity in the parent and doped BiS_2_-based superconductors still remain unresolved.

Moreover, one of the important problems in the layered systems is the inter- and intra-layer interactions. Similar to Fe-based superconductors, the interactions between superconducting BiS_2_ layers and blocking layers can be revealed *via* the nanoscale atomic displacements^17^,^18^. Hence, in order to understand the origin of superconductivity, it is critical to investigate the Eu valence and the local atomic displacements in the parent and doped Eu-containing BiS_2_-based superconductors.

The X-ray absorption spectroscopy (XAS), consisting of the X-ray absorption near edge spectroscopy (XANES) and extended X-ray absorption fine structure (EXAFS) spectroscopy, is an ideal technique to retrieve the substantial information of both valence transition and nanoscale atomic displacements, thus XAS has been widely applied in physics and chemistry^19^,^20^,^21^. For example, based on the “fingerprint effect”, Eu *L*_*3*_-edge XANES for EuFe_2_As_2_ presents the visually experimental evidence for the pressure-induced valence changes of Eu ions^22^. In addition, Bi *L*_*3*_-edge EXAFS were performed to probe the local atomic structure of BiS_2_-based systems^18^. In this contribution, we investigated the local structure of EuBiS_2_F-based system as a function of Ce-doping by XAS, providing the atomic site-selective information of valence changes and nanoscale atomic displacements.

## Results

### Role of Eu valence changes in the parent and Ce-doped EuBiS_2_F

**Figure 1:**
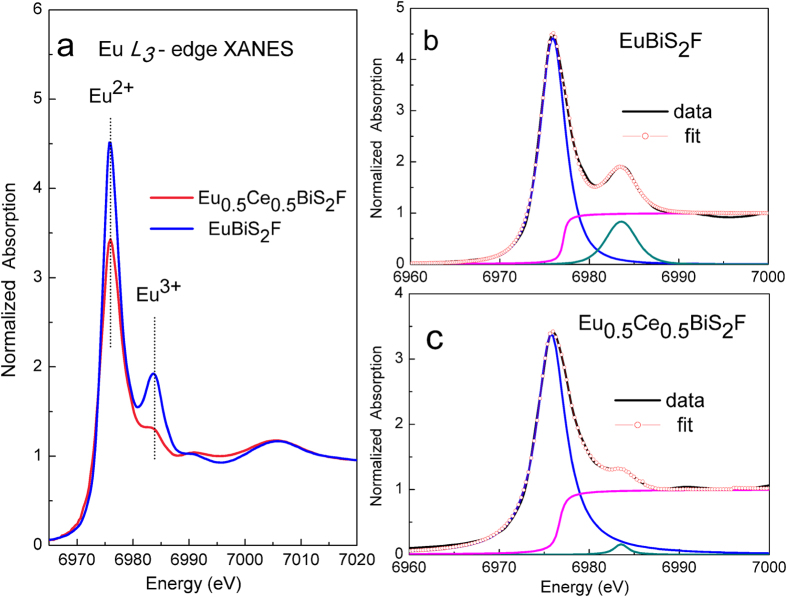
Eu *L*_*3*_-edge XANES spectra and curve fitting for EuBiS_2_F and Eu_0.5_Ce_0.5_BiS_2_F. (**a**) Normalized Eu *L*_*3*_-edge XANES spectra for EuBiS_2_F and Eu_0.5_Ce_0.5_BiS_2_F; (**b**) curve fitting for EuBiS_2_F; (**c**) curve fitting for Eu_0.5_Ce_0.5_BiS_2_F. The solid black line and red open circles correspond to the experimental data and the best fit, respectively.

For the Eu-containing superconductors, detailed investigations of the Eu valence change may provide valuable information on the electronic structure, which is fundamental for a better understanding of their superconductivity^22^,^23^. Figure 1a shows normalized Eu *L*_*3*_-edge XANES data for EuBiS_2_F and Eu_0.5_Ce_0.5_BiS_2_F. The main peak (6975 eV) and the other feature (6983 eV) in the Fig. 1a are associated respectively to Eu^2+^ (4*f*^7^) and Eu^3+^ (4*f*^6^)^22^.

Now we determine quantitatively the valence of Eu for the parent and Ce-doped EuBiS_2_F by fitting the XANES spectra to an arctangent step function and a Lorentzian peak for each valence state. The mean valence was determined by using a widely used method^24^,^25^:


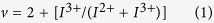


where *I*^2+^ and *I*^3+^ is integrated intensity of peaks corresponding to Eu^2+^ and Eu^3+^ on XANES spectrum. Based on the best curve fit in Fig. 1, we estimated the mean valence of Eu ions in EuBiS_2_F is +2.16(1), instead of +2, demonstrating the self-electron-doping nature in parent compound without any extrinsic doping. The mean valence of Eu in Ce-doped EuBiS_2_F is +2.05(1), basically consistent with previous crystallographic and magnetic structure data^15^. Therefore, these data confirm the Eu valence change, suggesting a potential relationship between the Eu valence and superconductivity.

**Figure 2:**
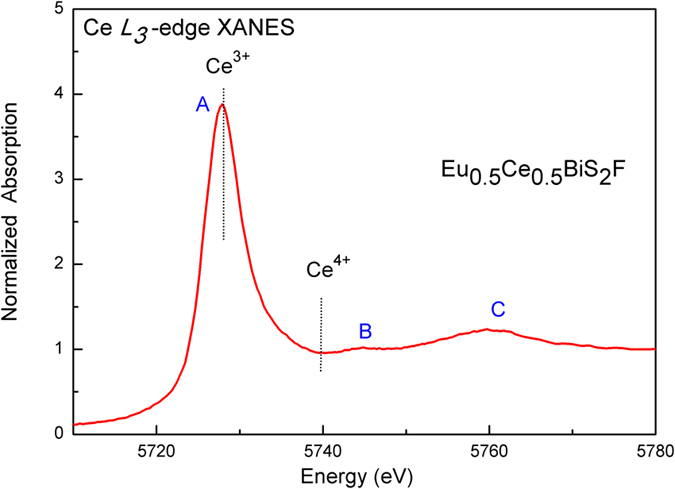
Normalized Ce *L*_*3*_-edge XANES data for Eu_0.5_Ce_0.5_BiS_2_F.

In Fig. 2 we focus on the normalized Ce *L*_*3*_-edge XANES in Eu_0.5_Ce_0.5_BiS_2_F, in which three main structures A, B and C can be identified. The first peak A around 5728 eV is associated to the transition from the Ce 2*p* core level to the vacant Ce 5*d* state mixed with the Ce 4*f* 1 final state, i.e. Ce^3+^ state^26^. On the other hand, the weak feature B around 5745 eV is a characteristic feature of layered rare-earth systems^26^, and its intensity is generally sensitive to the F atom order/disorder in the Eu/CeF layers. The third peak C is the so-called continuum resonance, providing the information on the local lattice structures. It should be noted that the energy difference between the characteristic Ce^3+^ (4*f*^1^) and Ce^4+^ (4*f*^0^) absorption peaks is approximately 12 eV, which is independent and is mainly determined by the Ce 2*p-*4*f* Coulomb interaction^26^. But in Fig. 2 we found no obvious evidence of Ce^4+^ feature around 5740 eV, demonstrating that the Ce valence in the Eu_0.5_Ce_0.5_BiS_2_F sample is essentially trivalent. Considering the valence of Eu, 50 at.% Ce-doping could cause an increment of mean valence for Eu/Ce ions, which increases from +2.16 of parent EuBiS_2_F to +2.53 of Ce-doped system. Consequently, additional 17% charges were induced upon Ce-doping in EuBiS_2_F, which is believed to be crucial for the superconductivity enhancement.

### Nanoscale atomic displacements in EuBiS_2_F and Eu_0.5_Ce_0.5_BiS_2_F

**Figure 3:**
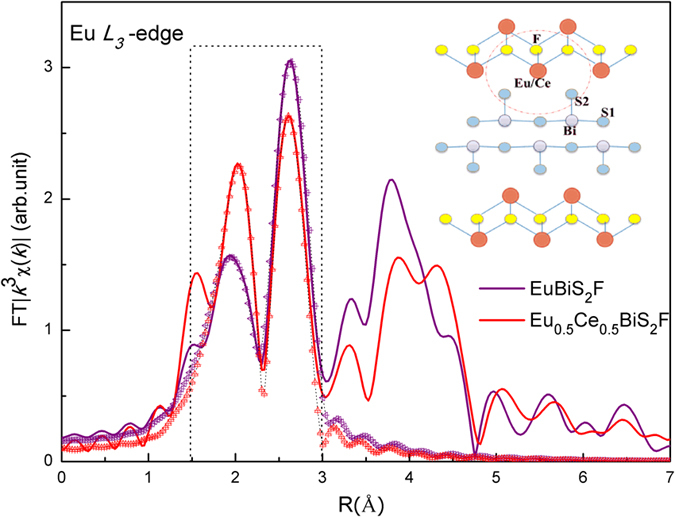
Fourier transform (FT) magnitudes of the Eu *L*_3_-edge EXAFS measured on EuBiS_2_F and Eu_0.5_Ce_0.5_BiS_2_F. Models fits to the FTs are also shown as triangles. The inset shows the local coordinate atomic clusters around Eu in cross-section view.

**Figure 4:**
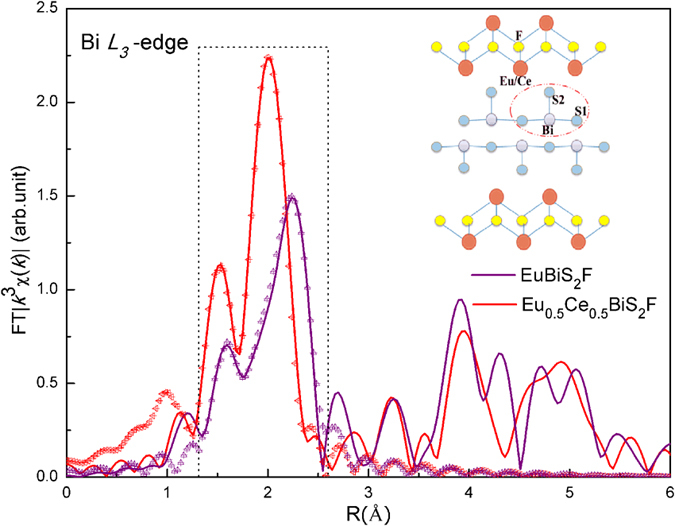
Fourier transform (FT) magnitudes of the Bi *L*_3_-edge EXAFS measured on EuBiS_2_F and Eu_0.5_Ce_0.5_BiS_2_F. Models fits to the FTs are also shown as triangles. The inset shows the local coordinate atomic clusters encircled around Bi in cross-section view.

As is well known, material properties are in a close relationship with its nanoscale atomic structure. Analogous to cuprates and Fe-based superconductors, Ce impurity could alter the local atomic displacements of both blocking layers and BiS_2_ superconducting layers. Therefore, to gain an insight into the atomic displacements induced by Ce-doping, we have undertaken detailed structural study by means of Eu and Bi *L*_*3*_-edge EXAFS measurements. Figures 3 and 4 display the Fourier transform (FT) magnitudes of the EXAFS oscillations providing real space information at Eu and Bi *L*_*3*_-edge, respectively. We have to underline that the positions of the peaks in the FT are shifted a few tenths of Å from the actual interatomic distances because of the EXAFS phase shift^27^. In the BiS_2_ layer the in-plane and out-of-plane S atoms are denoted as S1 and S2, respectively. The Eu atom is coordinated with four nearest F atoms at ~2.52 Å and four S2 atoms at ~3.04 Å. Therefore, the broad structure (*R* = 1.5~3.0 Å) in the FT of Eu *L*_*3*_-edge EXAFS corresponds to the contributions of Eu-F and Eu-S2 bonds. On the other hand, the near-neighbor of Bi atoms are one out-of-plane S2 atom at ~2.50 Å and four in-plane S1 atoms at ~2.87 Å. Therefore, the broad structure (*R* = 1.4~2.6 Å) in Fig. 4 contains information on the Bi-S2 and Bi-S1 bonds. Obviously, large changes in the FTs of both Eu and Bi *L*_*3*_-edge can be seen with Ce-doping, indicating the atomic displacements in blocking layers and also in the electronically active BiS_2_ layers.

The EXAFS amplitude depends on several factors and is given by the following general equation^28^:


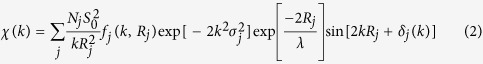


where *N*_*j*_ is the number of neighboring atoms at a distance *R*_*j*_, 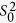 is the passive electron reduction factor, *f*_*j*_(*k, R*_*j*_) is the backscattering amplitude, *λ* is the photoelectron mean free path, *δ*_*j*_(*k*) is the phase shift and 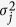 is the correlated Debye-Waller factor.

In order to obtain quantitative results, we firstly fit the peaks of EXAFS spectra at Eu *L*_*3*_-edge involving contributions of four Eu-F and four Eu-S2 bonds, which were isolated from the FTs with a rectangular window. The range in *k* space was 3~12 Å^−1^ and that in *R* space was 1.5~3.0 Å. Considering the absorption energy at Eu *L*_*3*_ (6977 eV) and *L*_*2*_-edge (7617 eV), the maximum wave-vector *k* for Eu *L*_*3*_-edge EXAFS is up to 12 Å^−1^. The spatial resolution 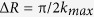
28 is about 0.13 Å with the *k*_*max*_ = 12 Å^−1^, which is sufficient to distinguish between Eu-F and Eu-S2 bonds. For the least-squares fits, average structure measured by diffraction on EuBiS_2_F system^13^ is used as the starting model. The backscattering amplitudes and phase shift were calculated using the FEFF code^29^. Only the radial distances *R*_*j*_ and the corresponding 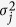 were allowed to vary, with coordination numbers *N*_*j*_ fixed to the nominal values. The passive electrons reduction factor 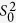 and photoelectron energy zero *E*_0_ were also fixed after fit trials on different scans. The best values for the 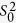 were found to be 0.9 and fixed to this value for all the shells. The number of independent parameters which could be determined by EXAFS is limited by the number of the independent data points *N*_ind_~(2Δ*k*Δ*R*)/π, where Δ*k* and Δ*R* are respectively the ranges of the fit in the *k* and *R* space^28^. In our case, *N*_ind_ is 8 (Δ*k* = 9 Å^−1^, Δ*R* = 1.5 Å), sufficient to obtain all parameters.

As shown in Table 1, upon Ce-doping the distance of Eu-S2 bond is essentially unchanged within the errors, while the Eu-F distance becomes slightly elongated from 2.51(1) Å to 2.54(1) Å, suggesting a thicker EuF layer induced by Ce-doping. Now we resort to the bond valence sum^30^ of Eu (Eu-BVS) using the formula 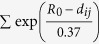, where *R*_0_ is an empirical parameter (2.04 and 2.53 Å for Eu-F and Eu-S bonds^30^, respectively) and *d*_*ij*_ denotes the measured bond distances between Eu and coordinate anions. Here, eight coordinate atoms (four F and four S2 atoms) were considered. Considering the bondlengths achieved from EXAFS fitting, the Eu-BVS value are +2.14(2) and +2.07(2) in EuBiS_2_F and Eu_0.5_Ce_0.5_BiS_2_F respectively, essentially in agreement with the valence information retrieved from our XANES data.

Meanwhile, Ce-doping also affects the local atomic structure of superconducting BiS_2_ layers. In Fig. 4 the broad peaks at Bi *L*_*3*_-edge were modelled by two shells, involving contributions of one Bi-S2 and four Bi-S1 bonds, which were isolated from the FTs with a rectangular window. The range in *k* space was 3~15 Å^−1^ and that in *R* space was 1.4~2.6 Å. Spatial resolution 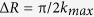 is about 0.10 Å, while the number of independent parameters *N*_ind_ is 9, sufficient to distinguish between Bi-S2 and Bi-S1 bonds and obtain all parameters.

Recently, it was reported that the enhancement of in-plane chemical pressure is responsible for the superconductivity in BiS_2_-based compounds^31^. Upon Ce-doping the sharp contraction of the in-plane Bi-S1 bond (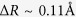, *i.e.* a higher in-plane chemical pressure) results in an enhancement of the packing density of Bi and S1 ions within the superconducting plane, which would enhance the hybridization of Bi 6p_x_/6p_y_-S 3p orbitals and result in an increase of *T*_c_. In addition, the fact that in-plane Bi-S1 bondlength decreases with Ce-doping, while the Bi-Bi distance (*i.e. a*-axis, from 4.0508(1) to 4.0697(1) Å) showing a small increase, indicating the puckering and large in-plane disorder of the Bi-S1 layer. Further information on the atomic disorder can be provided by the correlated Debye-Waller factors (σ^2^), measuring the mean square relative displacement (MSRD) of the photoabsorber-backscatterer pairs^32^. Data point out that the σ^2^ for the in-plane Bi-S1 distance in EuBiS_2_F is anomalously large, demonstrating a large configurational disorder within the Bi-S1 plane. Here, it is worth recalling that the large configurational disorder in BiS_2_ plane is quite common in BiS_2_-based superconductors, consistent with the anomalously large diffraction thermal factor of in-plane S1 atom^33^. Upon Ce-doping, the σ^2^ for the Bi-S1 bond reduces by 25% with respect to the parent compound, demonstrating that puckering of the Bi-S1 layer seems to be getting reduced; that is to say, a flatter Bi-S1 plane is also responsible for a higher *T*_c_. By contrast, the σ^2^ for the Bi-S2 bond is quite small and remains unchanged upon Ce-doping, indicating robust Bi 6p_z_-S 3p hybridizations. All these results suggest that Ce-doping can effectively tune the atomic displacements of BiS_2_ superconducting layers.

## Discussion

The Ce-doping effect on the valence state and local atomic displacement in the EuBiS_2_F system is investigated by using XAS measurements. First of all, the valence of Eu ions in EuBiS_2_F is estimated to be about +2.16(1), demonstrating the self-electron-doping nature without any extrinsic doping. Upon 50 at.% Ce-doping, the mean valence of Eu reduces to +2.05(1) and that of Ce ions are essentially trivalent. The main effect of Ce-doping is to provide additional 17% electrons into the system, beneficial for the superconductivity enhancement. The local atomic displacements can be revealed by Eu and Bi *L*_*3*_-edge EXAFS: 1) the in-plane Bi-S1 distance is characterized by a large configurational disorder in EuBiS_2_F-based system, which is quite common in BiS_2_-based superconductors; 2) both the shortening of the in-plane Bi-S1 bond (*i.e.* a higher in-plane chemical pressure) and the flatter Bi-S1 plane are responsible for an enhancement of superconductivity.

In summary, we established a relationship between Ce-doping and the *T*_c_ enhancement in EuBiS_2_F-based superconductors, in terms of valence changes and nanoscale atomic displacements. The new findings are promising for providing insights on the interplay of charge, local structure and superconductivity.

## Methods

Polycrystalline compounds of EuBiS_2_F and Eu_0.5_Ce_0.5_BiS_2_F were synthesized by solid-state reaction method^13^,^15^. The samples were well characterized for their phase purity, superconducting and other properties prior to the XAS measurements. The XAS spectra were collected at the BL-14W1 beamline of Shanghai Synchrotron Radiation Facility (SSRF). The storage ring was working at electron energy of 3.5 GeV, and the maximum stored current was about 250 mA. The energy of the incident energy was tuned by scanning a Si (111) double crystal monochromator with energy resolution about 10^−4^. The XAS spectra at Ce *L*_*3*_-edge, Eu *L*_*3*_-edge, and Bi *L*_*3*_-edge were collected with several scans in transmission mode at room temperature. Data reduction was performed using the IFEFFIT program package^34^.

## Additional Information

**How to cite this article**: Cheng, J. *et al*. Role of valence changes and nanoscale atomic displacements in BiS_2_-based superconductors. *Sci. Rep.* **6**, 37394; doi: 10.1038/srep37394 (2016).

**Publisher’s note:** Springer Nature remains neutral with regard to jurisdictional claims in published maps and institutional affiliations.

## Figures and Tables

**Table 1:** The fitting result at Eu and Bi *L*_*3*_-edge EXAFS upon Ce-doping.

System		EuBiS_2_F	Eu_0.5_Ce_0.5_BiS_2_F
Eu-F	*R* (Å)	2.51(1)	2.54(1)
	σ^2^(10^−3^ Ǻ^2^)	15.1(1)	12.5(2)
Eu-S2	*R* (Å)	3.04(2)	3.03(1)
	σ^2^(10^−3^ Ǻ^2^)	10.3(2)	12.6(1)
Bi-S2	*R* (Å)	2.49(2)	2.48(1)
	σ^2^(10^−3^ Ǻ^2^)	2.5(3)	2.6(1)
Bi-S1	*R* (Å)	2.79(2)	2.68(1)
	σ^2^(10^−3^ Ǻ^2^)	31.2(2)	23.4(1)

The errors represent maximum uncertainty, determined using correlation maps between different parameters and by analysing different EXAFS scans.
